# Toward Realigning Automatic Speaker Verification in the Era of COVID-19

**DOI:** 10.3390/s22072638

**Published:** 2022-03-30

**Authors:** Awais Khan, Ali Javed, Khalid Mahmood Malik, Muhammad Anas Raza, James Ryan, Abdul Khader Jilani Saudagar, Hafiz Malik

**Affiliations:** 1 Department of Computer Science and Engineering, Oakland University, Rochester, MI 48309, USA; awaiskhan@oakland.edu (A.K.); mraza@oakland.edu (M.A.R.); jaryan3@oakland.edu (J.R.); 2 Department of Software Engineering, University of Engineering and Technology, Taxila 47050, Pakistan; ali.javed@uettaxila.edu.pk; 3 Information Systems Department, College of Computer and Information Sciences, Imam Mohammad Ibn Saud Islamic University (IMSIU), Riyadh 11432, Saudi Arabia; aksaudagar@imamu.edu.sa; 4 Department of Electrical and Computer Engineering, University of Michigan, Dearborn, MI 48128, USA; hafiz@umich.edu

**Keywords:** automatic speaker verification, audio forensics, anomaly detection, face masks, COVID-19, surgical, filtered N95, cloth face masks

## Abstract

The use of face masks has increased dramatically since the COVID-19 pandemic started in order to to curb the spread of the disease. Additionally, breakthrough infections caused by the Delta and Omicron variants have further increased the importance of wearing a face mask, even for vaccinated individuals. However, the use of face masks also induces attenuation in speech signals, and this change may impact speech processing technologies, e.g., automated speaker verification (ASV) and speech to text conversion. In this paper we examine Automatic Speaker Verification (ASV) systems against the speech samples in the presence of three different types of face mask: surgical, cloth, and filtered N95, and analyze the impact on acoustics and other factors. In addition, we explore the effect of different microphones, and distance from the microphone, and the impact of face masks when speakers use ASV systems in real-world scenarios. Our analysis shows a significant deterioration in performance when an ASV system encounters different face masks, microphones, and variable distance between the subject and microphone. To address this problem, this paper proposes a novel framework to overcome performance degradation in these scenarios by realigning the ASV system. The novelty of the proposed ASV framework is as follows: first, we propose a fused feature descriptor by concatenating the novel Ternary Deviated overlapping Patterns (TDoP), Mel Frequency Cepstral Coefficients (MFCC), and Gammatone Cepstral Coefficients (GTCC), which are used by both the ensemble learning-based ASV and anomaly detection system in the proposed ASV architecture. Second, this paper proposes an anomaly detection model for identifying vocal samples produced in the presence of face masks. Next, it presents a Peak Norm (PN) filter to approximate the signal of the speaker without a face mask in order to boost the accuracy of ASV systems. Finally, the features of filtered samples utilizing the PN filter and samples without face masks are passed to the proposed ASV to test for improved accuracy. The proposed ASV system achieved an accuracy of 0.99 and 0.92, respectively, on samples recorded without a face mask and with different face masks. Although the use of face masks affects the ASV system, the PN filtering solution overcomes this deficiency up to 4%. Similarly, when exposed to different microphones and distances, the PN approach enhanced system accuracy by up to 7% and 9%, respectively. The results demonstrate the effectiveness of the presented framework against an in-house prepared, diverse Multi Speaker Face Masks (MSFM) dataset, (IRB No. FY2021-83), consisting of samples of subjects taken with a variety of face masks and microphones, and from different distances.

## 1. Introduction

Around the globe, life has changed drastically since the emergence of COVID-19. Variants of COVID-19, Alpha, Beta, Gamma, and Delta, and now Omicron, are proliferating rapidly and have affected millions of people. Moreover, the pandemic has caused significant social and economic disruption in daily life. As a consequence, the corporate and educational sectors have been partially or fully closed for extended periods of time. To counter this, Public Health Officials (PHO) and the World Health Organization (WHO) recommend various safety precautions and Standard Operating Procedures (SOP); i.e., vaccination, the use of face masks, social distancing, an increase in sanitization, and others. Although the most effective way to protect ourselves is to get vaccinated, the WHO recently released a statement urging both fully vaccinated and non-vaccinated people to continue to wear masks and practice social distancing in order to protect themselves and others from the recent variants of COVID-19. Since the virus is constantly changing through mutation, many new variants are expected to occur in near future. Additionally, cases [1,2,3] have been reported where vaccinated people became infected with the Delta and Omicron variants of COVID-19, thus wearing face masks and maintaining social distance may be mandated for COVID prevention for the foreseeable future. Similarly, in the United States, the Center for Disease Control (CDC) has recommended the continued use of face masks in order to minimize droplet dispersion and aerosolization of the COVID-19 virus and its variants [4]. Therefore, along with the other precautions, face masks have proved effective for the prevention of the spread of COVID-19. Consequently, face masks are becoming the new normal, and an essential part of the daily routine, as countries all over the globe mandate the use of face masks in public and the workplace. The logical extension of these trends and findings is that the use of face masks may be the new paradigm for many years to come.

While the use of face masks has led to a decrease in the spread of COVID-19, it has been at the expense of accuracy in voice-enabled and facial recognition applications. Additionally, this pandemic has discouraged the use of touch-based thumb recognition as an authentication mechanism as a result of the implementation of COVID-19 SOP’s. Moreover, facial recognition applications have also experienced authentication difficulties due to the different types of user face coverings. As a result, face biometric-based verification and the use of touch-based thumb recognition has been less reliable during the COVID-19 pandemic. These facts have amplified the need for voice biometrics for the authentication of various applications, e.g., banking and home automation, as voice-based authentication systems are safe and less likely to cause transmission of the virus. Since there is no restriction on vocal interaction in following the social distancing SOP’s recommended by the WHO and PHO, ASV systems for authentication are being heavily employed during the pandemic. Current ASV Systems, however, are not well prepared to handle the artifacts introduced in audio signals due to the use of face masks. Further, there are intrinsic and extrinsic factors that cause undesirable variations in the speech signals produced while wearing a mask. Intrinsic variability refers to human factors in speech production, i.e., vocal effort, speaking rate, and emotion. Extrinsic variability refers to how the acoustic speech signal reaches the recognition system or a human listener. Transmission media and distance effects are examples of extrinsic variables which introduce both the environmental noise (surrounding signals) and channel distortion due to the microphone or transmission channel, e.g., a telephone line or Internet Protocol (IP) network. Prior to COVID-19, ASV systems used vocal samples of the speakers in order to perform user verification and did not need to account for distortion due to facial masks. In contrast, the use of face masks during the conversation is now widespread.

An obstacle between the ASV system and the speaker, e.g., different types of facial covering: surgical, N95, or cloth masks, a balaclava, or a niqab, can attenuate the vocal samples to a great extent, particularly as the distance from the microphone increases, e.g., during the use of a hands-free device while driving. Thus, ASV systems need a robust countermeasure capable of filtering out artifacts introduced by the use of face masks from speech signals in order to obtain accurate speaker verification. Additionally, extrinsic factors, along with the face masks, raise some potential research questions for pre-trained ASV systems: (1) What is the impact of different types of face mask on pre-trained ASV systems? As we know, different types of face mask cause distinct attenuation in vocal samples. Therefore, the type of facial covering must be analyzed utilizing a variety of masks, i.e., surgical, cloth, N95, etc. (2) Differing microphones used in ASV systems can impact vocal samples differently. Good quality microphones contain noise reduction systems that produce better quality vocals; lower quality microphones are not able to deal as effectively with environmental noise. What happens when a user attempts to authenticate with a different face mask and using a different microphone? Will the pre-trained ASV system be able to recognize that user? (3) Distance between the speaker and ASV systems must also be analyzed as audio samples experience attenuation over distance. For example, it is very common these days to make phone calls using a car’s embedded microphone while driving, and the differing distance may prove challenging for ASV systems. Additionally, users need to follow the social distance SOP’s under COVID-19 precautions, which may introduce distance-based artifacts.

It is therefore necessary to investigate what happens when a registered user utilizes a face mask, records from different distances, and uses a different microphone for recognition. Can a pre-trained ASV system recognize the user’s vocals with these variations?

**Figure 1:**
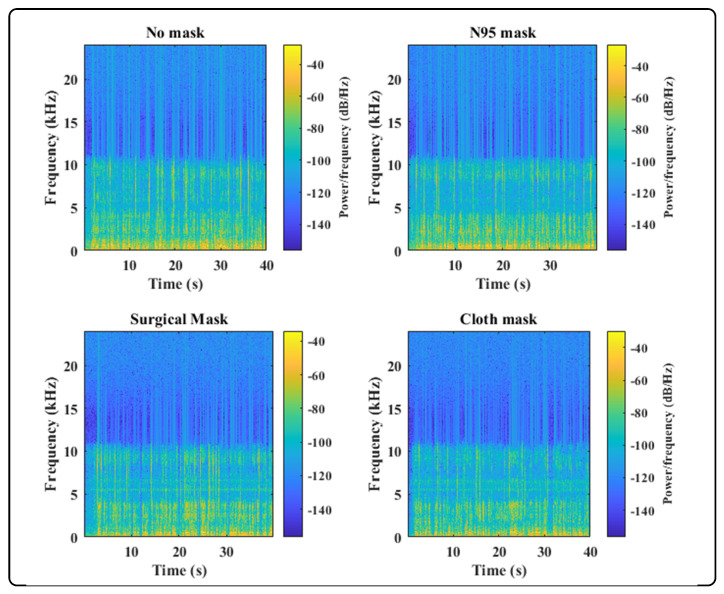
Spectrogram analysis of speech samples with and without a face mask.

**Figure 2:**
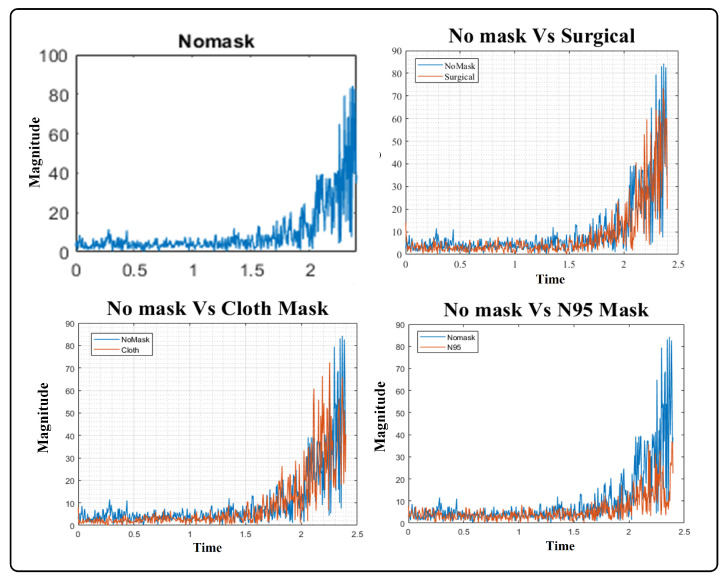
Comparison of different masked speech samples against samples without a mask.

These questions provided the motivation for the development of a solution to realign ASV systems. We performed a detailed analysis in which the subject’s speech sample, recorded without face masks and with three different face masks, including surgical, cloth, and filtered N95, was analyzed. For the initial experiment, we used voice samples from a single subject recorded in an indoor environment, i.e., a room. For each of the speech samples, the configuration and recording setup was the same, including the microphone and the distance between the microphone and speaker. We transformed the spectrum of speech signals using an FFT and plotted the signals in each time frame of the utterance, as shown in Figure 1 and Figure 2. We also plotted a speech sample without a mask against each of the masked samples in order to analyze the added distortion.

From the plots of speech samples and previous studies [5,6,7], we observed that the amplitude of cloth and N95 signals was adversely affected and a modest effect on the signal magnitude was perceived when the subject used a surgical face mask. Further, when the spectrum of each face mask was plotted against the spectra of no mask speech it was revealed that the cloth and N95 face masks suppressed signal amplitude significantly. Particularly, when the frequency was greater than 1 kHz, cloth and surgical face masks drastically attenuated the signal, while a N95 mask introduced moderate attenuation when frequencies were higher than 2 kHz. Moreover, cloth face masks modulate lower frequencies of the speech signal when the amplitude exceeds 1.5 kHz, as shown in Figure 1. These extensive variations in the speech samples motivated us to examine the performance of ASV systems with different face masks, microphones, and specified distances, and concluded that thorough experiments covering these variables are required. Additionally, there is a dire need to develop a robust ASV system that is capable of accurate user verification under these extrinsic circumstances; i.e., wearing a face mask, utilizing a different microphone, over distance.

This study has two main goals: First, to determine the effect of face masks on ASV systems which are trained on audio samples without masks, utilizing a single microphone and from a specified distance. Retraining ASV systems by using voice samples taken with face masks on is infeasible, therefore there is a need to make pre-trained ASV systems capable of recognizing speakers utilizing different face masks.

Our second goal is to develop a robust countermeasure to protect ASV systems by combating face mask effects in order to make them more effective for speaker verification. For this purpose, we need to analyze the impact of the variation and attenuation in vocal samples introduced by different masks. Additionally, mask impact varies from environment to environment (indoor and outdoor), microphone to microphone (iPhone microphone, Samsung microphone), and distance to distance (close to mouth, from 45 cm and 90 cm, such as in cars, etc.). These must also be analyzed. Previous studies in this domain have typically utilized small corpora collected under laboratory conditions. Moreover, fewer experiments and analyses were done in order to explore the effects of masks on acoustic signals. To the best of our knowledge, the impact of masks on ASV systems has not yet been studied under real-world conditions, such as those mentioned above. Similarly, different varieties of mask, (Cloth, Surgical, N95), have not been thoroughly examined to date.

This research focuses on the analysis of following factors in order to determine the effects of face masks under different circumstances:

- Detailed analysis of three commonly used face masks (surgical, cloth, N95) on audio signals.
- Performance analysis of ASV systems pre-trained on vocal samples without mask against those with masks.
- Impact of different face masks with different microphones collectively on ASV systems.
- Impact of different face masks and variable distances from the ASV systems (i.e., close to mouth in phone calls, from 1.5 feet ≈ 45 cm, and from 3 feet ≈ 90 cm in the car, etc.).

In this paper, after performing a detailed performance analysis of existing ASV systems we propose a new robust solution comprised of the following modules. The major contributions of the proposed work are:

- A novel feature extractor, “Ternary Deviated Overlapping Patterns (TDoP)”, is proposed and fused with the Mel Frequency Cepstral Coefficients (MFCC), and Gammatone Cepstral Coefficients (GTCC) for effective representation of input speech signals in order to achieve accurate speaker recognition.
- A robust anomaly detection model is presented to detect and segment the vocal samples into those with and without face masks.
- A filter-based approach is presented to exclude the impact of face mask from vocal samples segmented by the anomaly detection model.
- An improved and accurate ASV model with ensemble learning is presented that can verify speakers agnostic of face mask, from multiple microphones, and from variable distance without retraining.

The remainder of this paper is organized as follows: Section 2 provides a critical analysis of the existing literature. Section 3 includes the problem statement of this research study. Section 4 provides a discussion of the proposed method. Experimental results and analysis are presented in Section 5 to show the performance of the proposed method, and finally, the conclusion of the paper is provided in Section 6.

## 2. Related Work

There has been limited research on the impact of face masks on acoustic signals performed to date, and most of the prior work on face masks has focused on medical equipment only, e.g., surgical masks and N95 respirators. A summary of the prior work on the impact of face masks on ASV systems, along with the classification of masked and unmasked vocal samples, is presented below.

### 2.1. Effects of Masks on ASV System

Face masks add significant artifacts to speech, which in turn causes intelligibility problems. As a direct consequence, this attenuation has led to a decline in the performance of ASV systems during the current pandemic. Limited research has been done to examine the impact of face masks. In [8], Saeidi et al. analyzed the effects of four different face coverings: helmets, rubber masks, surgical masks, and hoods/scarves, on speaker recognition systems. The i-vector feature set was used, along with the Gaussian probabilistic linear discriminant analysis model, for speaker recognition. It was observed that a system trained on non-masked samples achieved a 91.88% average true positive rate, however this experiment was limited on the number of trials available for each mask type. Later on, Saeidi et al. [9] investigated the passive effect of masks on the sound of the speaker’s voice in ASV systems. Four mask types: surgical, scarf, motorcycle helmet and latex/Halloween, were used in this study. After determining the passive effect of each of these mask types on speaker audio, compensation for mask effects was done by applying a direct inversion with respect to the magnitude transfer function of the face mask. This compensation significantly improved the results on the verification of samples from latex/Halloween masks and motorcycle helmets, as these had the greatest impact on the speaker’s voice.

Face masks are known to affect the intelligibility of spoken words. Few analyses have been performed on the identification of spoken words under different mask conditions. Along this line, Loukina et al. [10] examined the impact of wearing face masks on the assessment of the spoken English language proficiency test. This study included data samples from 597 candidates who participated in the language proficiency test in Hong Kong. This test consists of a 20 min session comprised of four questions related to spontaneous speech. A total of 1188 audio samples were collected via headphones. These samples were given as input to an automated speech recognition system developed using the Kaldi toolkit. Experimental results revealed that face masks had a significant impact on speech patterns and the acoustic properties of the audio signal. To overcome this limitation, Ristea et al. [11] proposed an improved speech detection method for ASV systems in which the user was wearing a mask. Masked samples were transformed to simulate audio of the unmasked samples, and vice versa. This augmentation was done using cycle-consistent generative adversarial networks (GAN’s). Next, cycle-consistent GAN’s were trained on new samples which had been augmented to simulate the opposite type, i.e., masked for unmasked. The spectrogram obtained from the original and translated utterances were then fed to a residual neural network (ResNet) model with different depths, and these networks were integrated into an ensemble via an SVM classifier. When training the ResNet models with their proposed augmentations, a 0.9% improvement in average recall value was observed. This method achieved an unweighted average recall (UAR) of 74.6%, an improvement over the baseline model which achieved a UAR of 71.8%. However, this method is computationally more complex due to the need to run each audio sample through a cycle-consistent GAN.

### 2.2. Effects of Masks on Speech Acoustics

Face masks, other than transparent masks, prevent visual access to speaker lips, which in turn creates a barrier to communication. This in itself hinders speech perception, especially in noisy environments or when the listener possesses a hearing impairment. Recently, we have seen an exponential growth in studies analyzing the impact of face masks on speech. The authors of [12] conducted an analysis of the effect of surgical masks and noise on communication between healthcare professionals and patients. This study involved one talker and 15 listeners. Mendel et al. concluded in [12] that surgical masks did not create as much impact on speech understanding as the level of noise in a typical dental office. After that, Lamas et al. [13] conducted an investigation on the effects of different fabric face coverings on speech acoustics and intelligibility. This study concluded that surgical masks affected the acoustic signal significantly when compared to the following categories: no covering, niqāb, balaclava, handkerchief, scarf, fleece material, nylon stockings, and a loudspeaker cover fabric. It was also observed that surgical masks had an effect on frequency responses between 2.5–12.5 kHz and 14–24 kHz.

Limited research has been done to analyze the recognition of spoken words without the presence of background distortion. For this purpose, Fecher et al. [7] conducted a study to investigate audio only and audio-visual consonant recognition from speakers wearing different types of face coverings. This study, which was done in both the quiet and noisy conditions, found that participants used extra-oral facial cues to identify the consonants in spoken words. When face coverings obscure these movements, perception accuracy was adversely affected. Magee et al. [6] performed an acoustic analysis of speech using three common face mask types: N95, surgical, and cloth. The acoustic measures of audio under masks were analyzed using the timing, frequency, perturbation, and power spectrum of the signals. Attenuation at higher frequencies was reported i.e., above 3 kHz for N95 and 5 kHz for surgical and cloth masks. However, a solution to overcome these frequency fluctuations was not provided in this paper [6]. Corey et al. [5] also examined the effect of acoustic attenuation due to the surgical, cloth, and transparent face masks. The experimental results in [5] were different from those found in [6], as this study found all masks attenuate frequencies above 1 kHz. Further, it was observed that the attenuation was highest in front of the speaker. Moreover, face masks material also has an impact. The acoustic performance of transparent masks was worse when compared to surgical and cloth masks. In [14], Toscano et al. performed the recognition of spoken sentences in multi-talker babble with four different face masks including a surgical, N95 respirator, and two cloth masks. The impact of face masks on speech recognition was examined in the presence of background noise. Mean accuracy was calculated at two different noise levels (high and low) in order to determine the effect of the mask separately. For low level noises, no mask effect was observed, while in high level noise a drop of 18.2% in mean accuracy was reported.

### 2.3. Classification between Masked and Unmasked Audio

The classification of masked over unmasked speech is perhaps the most significant challenge yet to be addressed. Although limited research is available to observe the impact of face masks under some circumstances, the detection of the presence of a face mask from an audio sample is still a challenging task. Limited research was found on the classification of masked and non masked samples. In [15], Das et al. proposed a voice classification method based on the absence or presence of a face mask. For this purpose, audio features consisting of linear filter banks, instantaneous phase, and long-term information were used to capture the artifacts of speech with and without a face mask. More specifically, Linear Frequency Cepstral Coefficients (LFCC), Instantaneous Frequency Cosine Coefficients (IFCC), and Constant-Q Cepstral Coefficients (CQCC) were employed to train a Gaussian Mixture Model (GMM) for speech classification both with and without a face mask. The performance of this method was evaluated on the Mask Augsburg Speech Corpus (MASC), released by Compare in 2020. The MASC corpus is comprised of audio samples from 32 German speakers, both male and female, and has a total duration of 10 h. The fusion of these features, along with four baseline features, achieved an Unweighted Average Recall (UAR) of 73.5%. In [16], the authors conduct an in-depth analysis of the approaches submitted to the Mask SubChallenge (MSC) of the INTERSPEECH Compare challenge series. This challenge was conducted to determine whether a speaker was wearing a surgical mask or not via their audio speeches. The authors introduced the MASC database comprised of structured and unstructured text, and evaluated the top performing techniques submitted to the MSC challenge. A general classification framework comprised of specific, generic, features, and algorithm-based approaches was used to analyze the submitted approaches. Moreover, the authors identified three major factors that contributed to the effectiveness of the approaches in the challenge: ensemble learning, transfer learning, and data generation (augmentation). The best reported approach integrated a multi-band spectrogram with multiple CNNs, pretrained on imagenet. This approach achieved the highest UAR of 80.1%. Although this study primarily focused on the identification of a single type of face mask (surgical), the author emphasized the significance and necessity of addressing masks in voice-enabled biometrics systems. According to Kawase et al. [17], the impact of mask-wearing appears to arise from the loss of visual information that the brain employs to mitigate for auditory information deterioration. Some studies [18,19] investigated the acoustic consequences of wearing a mask. The affected frequencies were observed to range between 1 and 8 kHz, with the highest influence occurred between 2 and 4 kHz. These frequencies correspond to the ranges needed for voice biometrics, which are 1 kHz and 3–4.5 kHz [20].

A visual classification of face masks was also done using an LeNET model [21]. A Multi-Task Cascaded Neural Network (MTCNN) was used to detect the face region. The presented system classified the faces into two classes: masked face and unmasked face. Biometric verification of the masked and unmasked faces for user recognition achieved a 98.12% accuracy. In contrast, Patel et al. [22] used MFCC and Cochlear Filter Cepstral Coefficients (CFCC), along with instantaneous frequency features, to train a GMM to detect audio related attacks. The authors reported that the countermeasures relied more on the robust features rather than the classifiers.

Recently, Nguyen et al. [18] analyzed the acoustic characteristics of vocals with and without masks. Surgical and KN95 masks were used to determine the impact of face masks on acoustic signals. Five statistical measurements, including mean spectral levels, energy ratio, vocal intensity, Smoothed Cepstral Peak Prominence (CPPS), and harmonics-to-noise ratio were used to represent attenuation in the signal. The results of this study revealed that KN95 masks had a more significant impact than surgical masks. This work did not provide a solution to overcome the attenuation effect. On the other hand, instead of observing the various categories of face masks, Klumpp et al. [23] analyzed the impact of surgical masks only by presenting a method to classify the audio samples based on the existence of surgical masks. This classification uses phoneme recognition via two deep recurrence phonetic recognizer networks to determine if the speaker is wearing a mask or not. The first recognizer was trained on the German Verbmobi corpus, which contains 593 speakers, 307 female, and 286 male, and the recognizer was trained with 31 target phonemes, including silence. This recognizer was then used to compute phonetic labels for the audio in the MASC database. For each phoneme detected, it was assumed that it could have been produced either while wearing a mask or not, creating a target space of 61 total phonemes, including silence, which was expected to stay the same with or without a mask. This system achieved a UAR of 70.8%.

### 2.4. Face Mask Corpus Creation

The ASV community has developed a few corpora using audio samples where the speaker is masked in order to determine the impact of face masks. Natalie et al. [24] presented an audio-visual face cover corpus to examine speaker recognition under different mask conditions. This audio visual face cover corpus is comprised of high quality audio and video recordings of 10 native British English speakers wearing different types of face-wear: balaclava, hoodie/scarf, rubber mask, and surgical mask. According to this study, the impact of face-wear on acoustic signals is likely to stem from two different sources: the acoustic impedance characteristics of the mask material, and modification of the output signal due to face-wear’s interaction with the speech articulates and/or the air stream. Minor re-positioning will result in a rise in prominent acoustic and perceptual changes. It was concluded in [24] that face-wear should be treated as yet another factor causing inter-/intra-speaker variability in the “forensic trace” which is present in the context of the audio-video speech signal. To analyze different aspects of face masks, Fecher [24] created an Audio-Visual Face Cover Corpus (AVFCC), which includes audio and video of speakers with different masks and head-wear. The corpus consists of data from ten speakers, five male and five female, aged 21–36. All of the participants are native English speakers having South standard British accents. This corpus contains samples with eight types of face covering, among them surgical mask, hoodie and scarf combination, balaclava with lip hole and balaclava without lip hole, tape, and niqāb. The recording devices used were a headband microphone and two shotgun microphones. Two cameras were used, one in front, facing directly at the speaker, and another half profile camera off to the side.

Recently, a multi-domain English speech recognition corpus, GigaSpeech [25], with 10,000 h of high quality labeled audio was developed. The GigaSpeech corpus consists of five subsets of different sizes: 10 h, 250 h, 1000 h, 2500 h, and 10,000 h. The audio was first collected from audio books, YouTube and podcasts covering varieties of topics, e.g., arts, science, and sports, among others. Along with the dataset, a new forced alignment and segmentation pipeline was proposed in order to create the sentence segments suitable for speech recognition. Although this corpus covers the need for data samples for training and testing by deep learning approaches, it does not contain samples with face masks. Moreover, the existing corpus contains certain limitations in terms of the number of unique voices, microphone variability, environment specifications, and distance from the microphone to the subject.

## 3. Problem Statement

ASV systems used prior to the COVID-19 pandemic were trained on vocal samples without face masks. As a result of the pandemic, people use face masks to stay safe from COVID-19 and its variants. According to the WHO and the CDC, the spread of COVID-19 and its variants is expected to increase until 70% of the world population is vaccinated, necessitating a need for more robust ASV systems.

**Figure 3:**
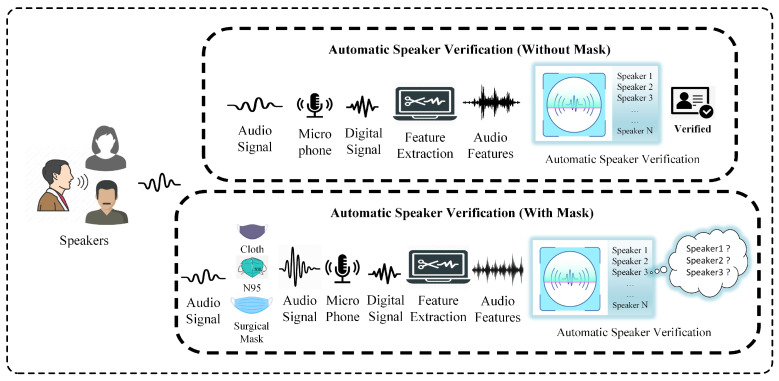
The impact of commonly used face masks on existing ASV systems.

Emergency vaccination programs around the globe are working tirelessly in an effort to vaccinate the majority of the world’s population but it will take some time due to challenges, among them resistance against vaccines, availability of vaccines, a limited number of approved vaccines, and a variety of complex sociopolitical issues. Recent studies have shown that vaccinated people may get infected by COVID-19 variants and can spread the virus to others. This highlights the importance of wearing face masks and maintaining social distance in order to protect others from COVID-19, so face masks may be the new reality for the foreseeable future. Thus, there is a desperate need to analyze the effects of face masks or facial coverings on pre-trained ASV systems. For example, are pre-trained ASV systems effective enough to recognize users wearing any type of face mask or covering, as shown in Figure 3? Additionally, other factors, i.e., microphone, environment, and speaker distance must also be examined along with the effects of masking. To overcome the impact of face masks on vocal samples, a robust detection model is required in order to effectively segment vocals into those with and without masks. Since the retraining of existing ASV systems to accommodate users who may be intermittently wearing a mask is infeasible, a dire need exists to develop more robust ASV systems which are capable of recognizing users either with or without a face mask. Development of a face mask effect filtering technique and robust feature set to make pre-trained ASV systems more compatible is essential to this task.

## 4. Proposed Method

**Figure 4:**
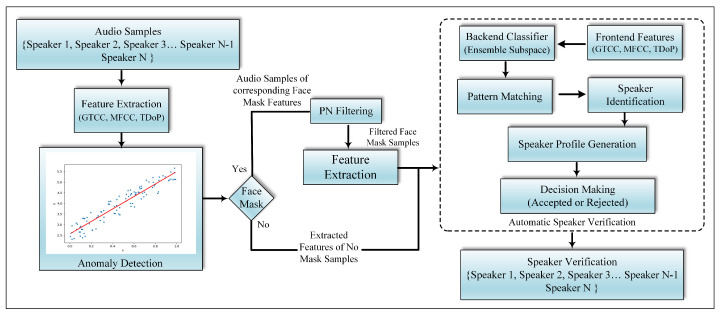
Proposed architecture for automatic speaker verification.

This paper provides a framework for the realignment of ASV systems in order to verify speakers with different face masks, including those who are unmasked, utilizing different microphones, and at varying distances from those microphones. To accomplish this, an effective feature descriptor is needed that is able to better capture the properties of vocal tract variations of different speakers with or without face mask. The effectiveness of local acoustic patterns in audio spoofing countermeasures for better capturing the traits of bonafide and spoof samples in [26] motivated us to develop the local Ternary Deviated overlapping patterns (TDoP) descriptor for ASV systems. For this purpose, we propose a 22D novel TDoP feature descriptor to better capture the vocal dynamics of different speakers in the time domain and fuse them with 14D GTCC and 14D MFCC features to extract the relevant information in frequency domain. We use the resulting 50D fused feature set in the following two modules: (A) Anomaly detection- trained on samples obtained from users without face masks on, this module detects speech utterances with face masks and sends these samples to the proposed Peak Norm (PN) filter, and (B) an ensemble classifier used to train and test the ASV model. Additionally, the identified samples classified as anomalous (i.e., with face mask on) by the anomaly detection module are filtered with a Peak Norm (PN) filtering solution to remove face mask effects, and external attenuation, i.e., microphones, distance, etc. The architecture of the proposed work is presented in Figure 4.

### 4.1. Feature Extraction

External factors, i.e., distance, microphone, and face masks, affect the acoustic properties of audio signals and cause performance degradation in speaker verification. In the proposed solution, we use a 50D fused features-set, including TDoP, GTCC, and MFCC, for anomaly detection, to detect face masked samples, and as an ASV classifier.

#### 4.1.1. Ternary Deviated Overlapping Patterns

**Figure 5:**
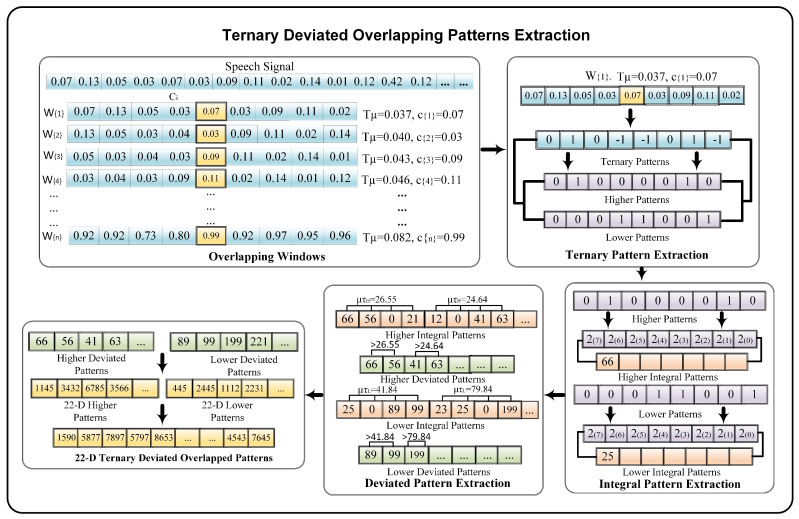
Extraction of the proposed Ternary Deviated Overlapped Patterns (TDoP).

We present the Ternary Deviated overlapping Patterns (TDoP) to extract integral features from speech samples. TDoP feature extraction consists of five phases that include overlapping windows, ternary, integral, and deviated pattern extraction, and 22D TDoP extraction. The overall process of TDoP feature extraction is shown in Figure 5. In the first phase, the input speech signal, consisting of *N* samples is divided into *i* = {1, 2, 3, …, *f*} overlapping windows $W^{\left(i\right)}$. Each $W^{\left(i\right)}$ contains 8 neighboring samples, along with a central sample $c_{i}$. In the overlapping windows, we use a step size of one, where each sample is selected as a central sample $c_{i}$ along with its left and right neighbors, as shown in Figure 5.

After this, we use three-level conditions to extract the ternary patterns from the obtained windows. For this purpose, instead of using manual thresholds, we compute an adaptive deviation threshold $T_{\mu }$ from each overlapped window $W^{\left(i\right)}$. The $T_{\mu }$ is computed as follows:

(1) $$T_{\mu }=\sqrt{\frac{\sum_{i=1}^{n}W^{\left(i\right)}\cdot {\left(\alpha_{i}-\Lambda \right)}^{2}}{N}}$$

where $\alpha_{i}$ and Λ refer to the sample and mean deviation of the window and *N* denotes the total number of samples. After obtaining the adaptive threshold $T_{\mu }$, we extract the ternary based patterns $\omega (W_{\left(n\right)}^{\left(i\right)},c_{i},T_{\mu }$) from each of the overlapped windows. For this purpose, we compute the three level thresholds using the center sample denoted as $c_{i}$ and the obtained threshold $T_{\mu }$. The three level thresholding includes two adaptive thresholds that vary from window to window of the speech signal. The pattern extraction is performed as follows:

(2) $$\omega \left(W_{\left(n\right)}^{\left(i\right)},c_{i},T_{\mu }\right)=\left\{\begin{array}{c} -1\;if\;W_{\left(n\right)}^{\left(i\right)}<LT_{\mu }\\ 0\;if\;LT_{\mu }\leq W_{\left(n\right)}^{\left(i\right)}\leq UT_{\mu }\\ 1\;if\;W_{\left(n\right)}^{\left(i\right)}>UT_{\mu } \end{array}\right.$$

where

(3) $$UT_{\mu }=c_{i}+T_{\mu }$$

(4) $$LT_{\mu }=c_{i}-T_{\mu }$$

$UT_{\mu }$ and $LT_{\mu }$ are the adaptive upper and lower thresholds of the window, *n* and *i* represent the neighboring samples and the overlapped windows, while $T_{\mu }$ denotes the deviation thresholds of the windows. The adaptive threshold $T_{\mu }$ controls the extraction of the ternary patterns. For instance, values in the audio samples depend on various factors, i.e., the surrounding environment, speaker voice, and volume, along with the devices used during recording. In this case, manual threshold selection without prior knowledge of the audio leads to an inaccurate extraction of the patterns. In contrast, adaptive thresholds, i.e., $T_{\mu }$, obtained through the actual sample values of the recording varies according to the specifications of each speech sample. Therefore, for ternary pattern extraction $\omega (W_{\left(n\right)}^{\left(i\right)},c_{i},T_{\mu })$ we define the lower $LT_{\mu }$ and upper $UT_{\mu }$ thresholds computed using the obtained adaptive $T_{\mu }$ and the central sample $c_{i}$. For instance, if the central sample $c_{i}$ has an integral value 0.07, the deviation threshold $T_{\mu }$ is obtained as 0.037, this makes the lower threshold $LT_{\mu }$ (0.07 − 0.037 = 0.033) and the upper threshold $UT_{\mu }$ becomes (0.07 + 0.037 = 0.107). For ternary patterns, the integral value 1 is assigned to the samples greater than the upper threshold 0.107 and −1 is used for the samples below the lower threshold. The values between the upper and lower threshold are replaced with the integral value 0 as shown in Figure 5.

Next, we extract the higher and lower patterns from the obtained ternary patterns of the signals. For this purpose, the obtained $\omega (W_{\left(n\right)}^{\left(i\right)},c_{i},T_{\mu })$ are further decomposed into a two-level representation, as shown in Equations (5) and (6).

(5) $$HW_{\left(n\right)}^{\left(i\right)}=\left\{\begin{array}{c} 1\;if\;\omega (W_{\left(n\right)}^{\left(i\right)},c_{i},T_{\mu })=1\\ 0\;\;Otherwise \end{array}\right.$$

(6) $$LW_{\left(n\right)}^{\left(i\right)}=\left\{\begin{array}{c} 1\;if\;\omega (W_{\left(n\right)}^{\left(i\right)},c_{i},T_{\mu })=-1\\ 0\;\;Otherwise \end{array}\right.$$

where $HW_{\left(n\right)}^{\left(i\right)}$ and $LW_{\left(n\right)}^{\left(i\right)}$ represent the higher and lower binary representation of the signal. In case of higher patterns $HW_{\left(n\right)}^{\left(i\right)}$, the value of 1 from ternary patterns $\omega (W_{\left(n\right)}^{\left(i\right)},c_{i},T_{\mu })$ remains unchanged while the rest of the values (−1,0) are replaced with the integer 0. Conversely, in the lower patterns $LW_{\left(n\right)}^{\left(i\right)}$, the value of −1 from the ternary features $\omega (W_{\left(n\right)}^{\left(i\right)},c_{i},T_{\mu })$ is replaced with 1 and the rest of the values (1,0) are replaced with 0. After that, the ternary-based patterns are converted into two levels, higher and lower, patterns with the numerical representation of positive integers (1,0). In the next phase, we extract the integral representation of the obtained non-negative higher and lower patterns. For this purpose, the extracted windows of 1’s and 0’s are converted into their equivalent decimal numbers as shown in Equations (7) and (8).

(7) $$I_{high}\left(W^{\left(i\right)}\right)=\sum_{n=7}^{n=0}Hp_{w}\left(W^{\left(i\right)}\right)\times 2^{\left(n-1\right)}$$

(8) $$I_{low}\left(W^{\left(i\right)}\right)=\sum_{m=7}^{m=0}Lp_{w}\left(W^{\left(i\right)}\right)\times 2^{\left(m-1\right)}$$

where $I_{high}\left(W^{\left(i\right)}\right)$ and $I_{low}\left(W^{\left(i\right)}\right)$ contain the integral representation of the higher $Hp_{w}$ and lower $Lp_{w}$ patterns. At this stage, we extract the two-level integral representatives of the audio signal. Although the integral patterns are extracted using three-level thresholds, the extracted higher and lower patterns still contain too many zero-valued patterns, which can affect the performance of speaker verification. To overcome this limitation, we employ a deviation threshold extraction on the obtained integral patterns $I_{high}\left(W^{\left(i\right)}\right)$ and $I_{low}\left(W^{\left(i\right)}\right)$. For this purpose, we select the $W^{\left(i\right)}$ windows with length l=4 and compute the adaptive deviated threshold $\mu \tau_{L}$ from $I_{low}\left(W^{\left(i\right)}\right)$ and $\mu \tau_{H}$ from $I_{high}\left(W^{\left(i\right)}\right)$ using Equation (2). The size of the window is decreased to capture more significant integral representatives. Next, we extract the values greater than the obtained deviation threshold. The rest of the values, along with all zero values below the threshold, are ignored in favor of the more robust deviated features. The extraction of the deviated integral features is shown in Equations (9) and (10).

(9) $$H_{TDoP}=I_{high}\;if\;I_{high}\left(W_{n}^{\left(i\right)}\right)>\mu \tau_{H}$$

(10) $$L_{TDoP}=I_{low}\;if\;I_{low}\left(W_{n}^{\left(i\right)}\right)>\mu \tau_{L}$$

where $\mu \tau_{L}$ and $\mu \tau_{H}$ represent the lower and higher deviation thresholds, *i* and *n* denote the window and neighboring samples, and $H_{TDoP}$ and $L_{TDoP}$ refer to the higher and lower non-zero deviated patterns. Finally, we obtain deviated integral patterns with the robust integral representation of the speech samples. In the last phase we compute the histogram of $L_{TDoP}$ and $H_{TDoP}$ patterns using Equations (11) and (12). The histograms of $L_{TDoP}^{n}$ and $H_{TDoP}^{n}$ are computed by applying the Kronecker Delta function. The Hist function, provided by MathWorks, is used to group the data into an equivalent number of bins.

(11) $$H_{TDoP}^{HT}=Hist(\sum_{n=1}^{22}\left(H_{TDoP}^{n}\right)$$

(12) $$L_{TDoP}^{HT}=Hist\left(\sum_{m=1}^{22}L_{TDoP}^{n}\right)$$

where $L_{TDoP}^{HT}$ and $H_{TDoP}^{HT}$ refer to the 22D lower and higher patterns of the speech signal and *n*, *m* represents the number of bins. $L_{TDoP}^{HT}$ and $H_{TDoP}^{HT}$ are the integral representatives of similar audio. Therefore, for the robust TDoP features, we added $L_{TDoP}^{HT}$ to the lower histogram $H_{TDoP}^{HT}$, as shown below.

(13) $$A_{TDoP}=H_{TDoP}^{HT}+L_{TDoP}^{HT}$$

where $A_{TDoP}$ represents the final 22D robust ternary deviated patterns. Our TDOP features analyze the audio frames locally by considering only 8 samples at a time, and employs the standard deviation based adaptive threshold approach while generating the ternary features, which makes them better able to capture even minor variation traits from the voice of different speakers, with or without a mask. Afterwards, we extract GTCC and MFCC to create a 50D fused features-set, which is used for speaker verification.

#### 4.1.2. Mel Frequency Cepstral Coefficients (MFCC)

Mel Frequency Cepstral Coefficients have proven very useful for various applications, i.e., voice recognition [27], speech recognition [28], and acoustic classification [29], and have become the de-facto standard for audio parameterization. They are perhaps the most extensively used speech features in the field of voice/speaker recognition [30,31,32]. MFCC features are able to extract perceptually significant elements of the speech spectrum, and this impressive performance is the motivating factor for using them in the proposed method. The acoustic characteristics of vocal samples vary constantly throughout the signal. In order to accurately extract MFCC features, we divide the audio signal into small time-scale frames F[t], which may also be referred to as windows. The length of the frame F[t] controls the sensitivity of the MFCC features. For instance, if the length of the window is short, there are not sufficient samples to extract reliable features. Conversely, in the case of long windows, the signal changes continuously throughout the frame. Therefore, after a detailed analysis, we divided the audio signal A[n] into *T* frames F[t] of length l=30 ms with an overlap of 15 ms. After framing, the Hamming Window Function (HWF) was then used to minimize signal discontinuity from the beginning and end of each extracted frame F[t]. Next, a Discrete Fourier Transform (DFT) was used to extract the power spectrum of the obtained windows. After that, a filter bank is used to extract the Mel-scales $M_{k}^{\left(FB\right)}$ of the DFT windows. Next, we extracted the log Mel-spectrum by applying a logarithmic function to the extracted Mel-scales. Finally, a Discrete Cosine Transform (DCT) was applied on the extracted log-spectrum in order to derive the MFCC features. The overall extraction of MFCC is shown below.

(14) $$A_{mfcc}=\sum_{j=1}^{k}\left(logM_{k}^{FB}\right)cos\left[\delta \left(k-\frac{1}{2}\right)\frac{\pi }{k}\right]$$

where $M_{k}^{\left(FB\right)}$ represents the Mel-scales, *k* and δ is the number of Mel-scales and spectrum. The resultant $A_{mfcc}$ contains the 14D MFCC coefficients of the speech samples.

#### 4.1.3. Gammatone Cepstral Coefficient (GTCC)

Gammatone Cepstral Coefficients are a linear-based filter specified by impulse responses and the product of gamma and sinusoid patterns. Taking as a basis the MFCC computation scheme, GTCC incorporates biologically inspired filters that have proven to be significant in auditory processing [33]. Gammatone function models the human auditory filter response. The authors of [34] reported a resemblance between the Gammatone filter’s impulse response and the one acquired from humans. According to the results reported, GTCC’s performance is much better than that of other state-of-the-art audio features. In addition, GTCC features are also known to be robust in noisy environments [34]. This exceptional performance of these bio-inspired features motivated us to assess their influence on speaker verification. For GTCC feature extraction, an input audio signal A[n] is divided into short windows for the Spectra temporal analysis with *N* samples as *n* = {1, 2, 3, …, *f*}, and W(n), 0<n≤N−1. The input signal A[n] is divided into 30 (Millisecond) ms short windows with an overlap of 15 ms as shown in Equations (15) and (16).

(15) $$W_{GT}^{\left(i\right)}=A[n]\times W(z)$$

(16) $$W\left(z\right)=\left(p-q\right)cos\left[2\pi \times \frac{z}{Z-1}\right]$$

where *p* and *q* represent the length and the size of overlapping windows, *z*, which denotes the number of windows, ranges between 0<z≤Z−1 and *Z* represents the total number of windows. A Fast Fourier Transform is applied on the obtained windows W(z) to emphasize the irrational signal frequencies. After that, a Gammatone (GT) filter bank, composed of frequency responses, is used to extract the energy sub-band of the transformed windows. Finally, a log function and DCT are employed on the resultant windows of the GT filter bank. The log and DCT functions are analogous to human loudness perception, correlate the logarithmic-compressed filter outputs, and obtain better energy compaction. The GTCC features are extracted as:

(17) $$A_{gtcc}={\frac{2}{\omega }}^{\left(1/2\right)}\sum_{\eta =1}^{\omega }log_{10}\left(X_{\eta }\right)cos\left[\frac{\pi \eta }{\omega }\left(\varphi -\frac{1}{2}\right)\right]$$

where $X_{\eta }$ is the energy of the η sub-band, ω refers to the number of Gammatone filters, and φ denotes the number of GTCC features. The obtained $A_{gtcc}$ contains the 14D GTCC features of the transformed audio signal.

In the next step, the integral features $A_{TDoP}$ and cepstral coefficients, including $A_{GTCC}$ and $A_{MFCC}$, are concatenated to create the 50D fused feature-set for speaker verification. The fused features are extracted as follows:

(18) $$A{\left[n\right]}^{f}=A_{GTCC}⌣A_{MFCC}⌣A_{TDoP}$$

where $A{\left[n\right]}^{f}$ represents the 50D fused features-set, $A_{GTCC}$, $A_{MFCC}$, and $A_{TDoP}$ refer to the GTCC, MFCC, and TDoP features, respectively, and ⌣ is the concatenation operator.

More specifically, $A{\left[n\right]}^{f}$ includes the 28D cepstral coefficients of GTCC and MFCC (14D each), and 22D integral TDoP features. The extracted feature set was used during the training and testing of the ASV system. In the proposed framework, the ASV system was trained on speech samples recorded without a mask, and testing is performed on the samples recorded using a face mask. We then employed an anomaly detection model, which detected and segmented the speech samples (with and without face mask) in order to train and test the ASV system. The proposed anomaly detection model is discussed below.

### 4.2. Anomaly Detection

The detection of the presence of a face mask in an audio sample is mandatory if the attenuation due to face masks in speech signals is to be mitigated. To do this, a linear regression-based anomaly detection model was proposed to segment the audio signal into two types, those recorded with a face mask and those without wearing a face mask. In the proposed framework, we trained the ASV model with the indicated features extracted from input speech signals recorded without face masks, while the testing was performed on signals recorded with a face mask. More precisely, during the testing of the ASV system, we employed the anomaly detection model to test the segmented samples without a face mask, while the samples where a face mask was detected were passed to the PN filter instead of the ASV classifier, as shown in Figure 4.

#### 4.2.1. Training

In the training phase, we used the extracted feature set of speech signals recorded without wearing a face mask to train our ASV system. Since TDoP features are integral, and GTCC and MFCC have fractional representation, we normalized all features using the min-max normalization shown below:

(19) $$A{\left[\bar{n}\right]}_{norm}=\frac{A{\left[n\right]}^{f}-A{\left[n\right]}_{min}^{f}}{A{\left[n\right]}_{max}^{f}-A{\left[n\right]}_{min}^{f}}$$

where $A{\left[n\right]}^{f}$ represents the set of extracted features, and $A{\left[n\right]}_{max}^{f}$ and $A{\left[n\right]}_{min}^{f}$ denote the maximum and minimum values. After the normalization process, all feature values lie in the range between 0 and 1. Next, a linear regression model was used for training the model, as shown in Equation (20).

(20) $$A{\left[n\right]}_{prid}=\alpha \times A{\left[\bar{n}\right]}_{norm}+\vartheta$$

where $A{\left[\bar{n}\right]}_{norm}$ denotes the normalized features, $\bar{n}$ is the total number of speech samples, and α and ϑ represent the slope and intercept of the extracted features. Next, an anomaly detection threshold was obtained using the Mean Squared Error (MSE) of the fused features. This threshold was used to differentiate signals with a face mask from those without a mask. We then computed the loss function $A{\left[\bar{n}\right]}_{\tau 1}^{NM}$ of the speech samples without face masks using the obtained $A{\left[n\right]}_{prid}$ as shown in Equation (21).

(21) $$A{\left[\bar{n}\right]}_{\tau 1}^{NM}=\frac{1}{n}\sum_{i=1}^{n}\left[A{\left[n\right]}_{prid}-{\left(A{\left[n\right]}_{norm}\right)}^{2}\right]$$

Next, we tested the regression model and segmented the speech samples into those with and without a face mask by using the obtained $A{\left[\bar{n}\right]}_{\tau 1}^{NM}$.

#### 4.2.2. Testing

In this phase, we tested the model on speech signals recorded with different face masks. For this purpose, first, the pre-processing of min-max normalization was applied to obtain the identical representation of the testing samples using Equation (19). After that, the loss function was computed using the mean squared error, as in the training of the model. Finally, the computed loss function from the testing samples was compared with the anomaly detection threshold under the following condition: if the loss function of the sample was higher than the anomaly detection threshold, the sample was considered to have an anomaly (face mask), while samples with a loss value below the threshold were categorized as samples recorded without a face mask.

(22) $$LF_{w}\left(W^{\left(i\right)}\right)=\left\{\begin{array}{c} W_{Mask}^{F}\;if\;A{\left[n\right]}_{\tau 1}^{NM}<A{\left[n\right]}_{\tau 2}^{mask}\\ W_{Mask}^{O}\;\;\;\;Otherwise \end{array}\right.$$

In the above equation, $W_{Mask}^{F}$ and $W_{Mask}^{O}$ denote the samples with and without face mask, respectively, $A{\left[n\right]}_{\tau 1}^{NM}$ represents the anomaly detection threshold, and $A{\left[n\right]}_{\tau 2}^{mask}$ denotes the loss threshold calculated from the audio samples. The samples with a loss value greater than the anomaly detection threshold are attenuated samples. The loss function is greater due to the presence of face masks and/or other extrinsic factors such as distance, microphone, etc. Using the anomaly detection model we are able to segment the speech samples into those recorded with and those without face masks.

### 4.3. Peak Norm Filtering

In this subsection, we present a solution to overcome the observed attenuation caused by face masks and external factors, i.e., distance and microphones. The spectral and time-domain analysis of the signals demonstrates that each type of variation and mask introduces a distinct distortion in the acoustic pattern, and the added distortion leads to deterioration of performance of the ASV system. To overcome this decline, we employ the Peak Norm (PN) solution to stabilize the speech signals recorded with distinct variation.

By convention, the magnitude of an audio signal can span the range between min=−1 and max=+1, so the maximum magnitude variation that can take place is 2. As we have shown, the magnitude of a signal is adversely affected by the presence of a face mask and/or external factors. To combat this, in the proposed solution we adjust the magnitude of the speech signals by bringing the Pulse-code modulation (PCM) value to the required level. For this purpose, an input audio signal is partitioned into *x* = {1, 2, 3, …, *w*} windows $W^{\left(x\right)}$ of 15 ms. After that, we applied an inverse FFT and extract the impulse responses of the signals, as shown in Equations (23) and (24).

(23) $$W^{\left(x\right)}=A[n]\times W(x)W^{\left(ir\right)}=FFT^{-1}\left(W^{\left(x\right)}\right)$$

where $W^{\left(x\right)}$ represents the window and $W^{\left(ir\right)}$ denotes the impulse response of the windows. After these transformations, we use pulse code modulation to extract the real part of the values from the obtained impulse responses. More specifically, during modulation the magnitude of the speech signal is continuously sampled at uniform intervals. After that, each of the obtained samples was further quantized to the closest sample within the interval range. In the next step, we computed the magnitude decibel (dB) values as shown below:

(24) $$W^{\left(pcm\right)}=\sum_{a=1}^{x}PCM\left[W^{\left(ir\right)}\right]$$

(25) $$W_{dBFS}^{\left(x\right)}=T\cdot \log_{10}\frac{W_{max}^{\left(pcm\right)}}{W_{min}^{\left(pcm\right)}}$$

where *T* has a value of 20, and $W_{max}^{\left(pcm\right)}$ and $W_{min}^{\left(pcm\right)}$ represent the maximum and minimum values of the windows. Next, we extract the peak threshold and bring the magnitude of the speech sample to the desired level, as shown below:

(26) $$FS_{Th}=\sum_{u=1}^{v}W_{F}^{\left(x\right)}\times 10^{\frac{k}{m}}$$

where *v* represents the number of samples, and *k* and *m* denote the required levels of restoration. In our case, we used k=−6 and m=20 because we obtained the best results utilizing these parameters. Finally, the magnitude of the speech samples is recovered using the peak threshold as shown in Equation (28).

(27) $$FS_{Filt}=FS_{Th}\times \frac{1}{max\left(abs\right)(W^{\left(ir\right)})}\times W_{dBFS}^{\left(i\right)}$$

In this equation, $FS_{Th}$ represents the peak threshold, abs refers to the absolute value, while $W^{\left(ir\right)}$ denotes the impulse responses of the signal. Finally, the resultant $FS_{Filt}$ includes the restored speech sample with the improved level of magnitude. In the next section, we report on the classifier used for the training and testing of the proposed system.

### 4.4. Classification with Ensemble Learning

In this phase, we use the extracted fused features and an ensemble classifier with subspace discriminate to train and test the ASV system. Different types of ensemble learning, e.g., bagging, boosting, and sub-spacing with reduced variance and bias, have proven significant in the training of weak classifiers. In particular, attribute bagging, also called subspace ensemble, performed adequately in signal processing, e.g., acoustic classification [29] and audio recognition [35]. More specifically, instead of the entire feature set, subspace bagging utilizes Linear discriminant Analysis (LDA) and Random Discriminant Analysis (RDA) to extract random low dimensions of the features. Moreover, random subspace features require less memory consumption and offer proficient handling of the missing and lower dimensions. In the current work, we use a bagging ensemble with subspace discriminate for training and testing of the ASV system. More specifically, the features are arranged using the Random Subspace Method (RSM) to train the weaker classifiers of the data. To analyze the performance of the proposed feature set, the ASV model is first trained on speech samples recorded without face masks. We start by classifying the speech samples into multi-classes i.e., $\phi_{\iota }$={class(a),class(b),class(c)⋯⋯,class(v)}. Next, ensemble learning with subspace discriminate is used to train and aggregate the output of each weak-learner in order to determine the final prediction. After that, the features of each class are divided into a subset of features using the RSM function. The randomly selected weak classifiers are then used, along with the obtained subset features, to predict the suitable class. Each weak classifier votes for the appropriate class. Accordingly, the final prediction of the class is obtained by aggregating the highest number of votes for the specific class. Finally, the ASV model is trained with the values predicted by the subspace modeling. The final output of the classifier is obtained through the equation shown below:

(28) $$class\left(\phi \right)=argmax_{\left(y_{i},\lambda ,C\right)}\left(\sum_{u=1}^{v}h\left(C_{\omega }\left(y\right),\lambda \right)\right)$$

In the above equation, ϕ denotes the class from {*a, b, c, …, v*}, *C* represents the subset features, ω is the classifier, and *h* denotes the predictor of the model. After training the model with the extracted features, as shown in Figure 4, detailed testing is performed to show the effectiveness of proposed framework and the results are reported in the next section.

## 5. Experiments and Results

### 5.1. Dataset

Performance of the proposed system was evaluated on our custom-developed Multi-Speaker Face Mask (MSFM) dataset. The MSFM dataset consists of the speech samples from 20 subjects, including 4 native United States (US) citizens (1 female, 3 males) and 16 non-natives (9 females, 7 males). The speech samples were recorded in real-time, indoor and outdoor environments, e.g., bedroom, kitchen, balcony, playground, etc. The speech samples were recorded in the English language, both with and without wearing face masks.

MSFM subjects employed three different types of face masks while recording: surgical, cloth, and N95. In addition, the MSFM dataset includes the speech samples from different microphones (wired, wireless, cell phone), made by different manufacturers (iPhone, Samsung, Huawei, Redmi, Infinix), recorded at three different distances (close to mouth, from 45 cm, and from 90 cm). The MFSM dataset was prepared after institutional review board (IRB) approval (IRB No. FY2021-83) at Oakland University. The MSFM dataset consists of 692 speech samples with a total length of 11 h 54 m, at a sampling rate of 48 kbps.

Specifically, 326 speech samples were recorded without wearing a face mask and 366 samples were recorded with the three different face masks (surgical, cloth, and N95). Also, 200 speech samples were recorded with a single microphone and 63 samples were recorded with multiple microphones (wired, wireless, cell phone). In addition, 126 speech samples, 63 each, were recorded at a distance of 45 cm and 90 cm from the microphone. For the speech samples with face masks, 240 samples were recorded with a single microphone and close distance, 63 samples were recorded with multiple microphones and 126 samples were recorded from the 45 cm and 90 cm distances, as mentioned in Table 1.

The speech samples were further sub-clipped into 2000 sub-samples without face masks and 1000 with face masks in order to overcome the limitation of training samples for the anomaly detection model. Speech samples with three different face masks, multiple microphones, and varying distances make the MSFM dataset more challenging for the ASV system. To the best of our knowledge, no existing corpus involves as much variation in the data used for the speaker verification.

### 5.2. Performance Comparison using Baseline GMM Classifier

The Gaussian mixture model (GMM) is a probabilistic model that represents the presence of subsets with parametric estimates using a finite number of Gaussian distributions. The GMM has been shown to be effective in a variety of acoustic applications. For instance, in the most recent ASVSpoof2021 [36] competition and preceding challenges, a GMM was used as the baseline classification model [37,38,39], and a GMM is employed in speaker verification using fronted acoustic features, such as MFCC [39]. Therefore, as described in [39], we examined speech samples using well-known MFCC features and GMM-based classification to evaluate how face masks influenced existing ASV systems. In order to assess speech samples, the entire speech signal recorded without face masks is trained using the GMM model. The parameters for each speaker are then estimated using the Maximum Likelihood Method (MLM). For fitting the mixture-of-Gaussian models, we used the expectation-maximization (EM) approach in testing. We implemented the GMM model with the expectation-maximization (EM) algorithm provided by the scikit-learn organization [40]. During the GMM model’s training, we employed diagonal co-variances and 9 components with 20 iterations. In the testing of speech samples without a face mask, the GMM model obtained an accuracy of 98.7%. Conversely, when we evaluated the model with speech samples recorded with three different face masks (surgical, cotton, and N95), the model’s accuracy dropped to 69%. Thus, the accuracy of the GMM model was shown to drop by approximately 30% when face masks were used during the recording of the speech samples. By this experiment we can infer that the traditional acoustics features and baseline GMM classification [39] failed to perform speaker verification when the speaker used face masks. This indicates that current ASV systems need to be re-aligned.

### 5.3. Performance Evaluation of the Proposed System

Performance of the ASV system was evaluated using the error rate, precision, recall, accuracy, F1-score, Kappa index, Matthews Correlation Coefficient (MCC), and Classification Success Index (CSI). We trained the ASV system, with the proposed features, to identify subjects with their speech samples. We used speech samples obtained without wearing a face mask for training and testing of the ASV system. The ASV system was also tested against the speech samples recorded by users wearing different face masks in order to show the impact of face masks on ASV systems. We designed a multi-stage experiment to test the ASV system against the speech samples recorded with multiple microphones (wired, wireless, cell phone) and fixed distance. In the first stage, we tested the ASV system on audio samples with and without face masks, recorded with multiple microphones. In the second stage, the ASV system was evaluated against speech samples with and without face masks, recorded at a 45 cm and 90 cm distances between the subject and the microphone.

We employed the proposed features to train different ensemble classifiers and obtained the best results with ensemble subspace discriminant. We employed the ensemble subspace discriminant classifier in the proposed work. We tuned our ensemble classifier on 30 distinct learners and 21 subspace dimensions and achieved the highest accuracy 0.96 and UAR 0.98 with these parameters. The results are reported in Table 2. All of the ensemble classifiers were analyzed for accurate subject verification. For the speech samples without face masks, the proposed system, using the ensemble subspace discriminant classifier, achieved an accuracy of 0.99. Similarly, for the speech samples recorded with different face masks, the proposed system achieved an accuracy of 0.92. We achieved the accuracy of 0.63, 0.25, 0.47, and 0.69 using a Bagged tree, Subspace KNN, Rusboosted, and Boosted trees, respectively. These results demonstrate the significance of a subspace discriminate classifier on ASV. Moreover, our system achieved the lowest error of 0.01, and precision, recall, and F1-scores of 0.99, Kappa and CSI of 0.98, and MCC of 0.99 for the audio samples without face masks. Further, we achieved an error rate of 0.08, Kappa of 0.86, MCC 0.90 and CSI of 0.98 for the audio samples recorded with one of the three face masks.

**Figure 6:**
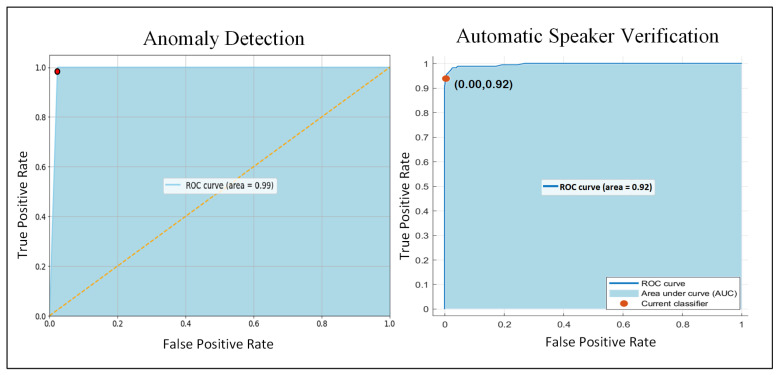
ROC curves of the anomaly detection and ASV system.

The Receiver Operating Characteristic (ROC) of the proposed system for anomaly detection and ASV is presented in Figure 6. From these ROC curves, we can conclude that our system offers a remarkable performance for anomaly detection and ASV.

### 5.4. Performance Comparison of Standalone and Fused Features

We tested the performance of TDoP, GTCC, and MFCC features, both individually and in their fused combinations for ASV, against speech samples recorded with and without face masks. For this, we tested each of the descriptors, and their fusion, on the ensemble subspace discriminate classifier and the results are shown in Table 3. Despite the fact that MFCC features were the most widely employed and retrieved perceptually significant elements of the speech spectrum, they failed to perform effectively due to the presence of noisy speech samples in the proposed MSFM dataset. Similarly, GTCC simulates human auditory filter responses, which have been shown to be resilient to noise. However, capturing biological aspects is insufficient when the dataset (like MSFM) has too much diversity, i.e., various microphones, varied distances, and multiple face masks. On the contrary, the proposed TDoP features by employing the standard deviation based adaptive thresholding scheme successfully captures the significant characteristics from the speech signal containing dynamic variations. It is important to mention that we also tested the feature fusions along with the standalone features, and the fused TDoP, GTCC, and MFCC features produced the best results in speech verification. Due to the high variability in the MSFM dataset, i.e., environment, microphone, and distances, the fused features-set consisting of TDoP, GTCC, and MFCC outperforms the standalone features as well as other combinations. Thus, we employed the TDoP-GTCC-MFCC fused features-set in the proposed work. These results show that the fused TDoP, GTCC, and MFCC features achieved the best results in speech verification. It can also be observed that due to the significant diversity in the MSFM dataset, i.e., environment, microphone, and distances, standalone features do not perform well as the fused features. However, the fusion of TDoP with GTCC and MFCC achieved the best results on all of the combinations. This led us to choose the TDoP-GTCC-MFCC features fusion for this work. Our fused features (TDoP-GTCC-MFCC) achieved an accuracy and precision of 0.99, recall of 0.97, F1-score of 0.98, and an error rate of 0.01 on the samples recorded without a mask, and achieved an accuracy and precision of 0.91, recall of 0.92, F1-score of 0.91, and an error rate of 0.08 on the speech samples recorded with different face masks.

We also observed deterioration in the results when tested on the speech samples with masks. An increase of 7%, 5%, 12%, 9%, 5%, 7%, and 7% in the error rate was observed for TDoP, GTCC, MFCC, TDoP-GTCC, TDoP-MFCC, MFCC-GTCC, and TDoP-MFCC-GTCC, respectively. Additionally, we evaluated the performance of the standalone and fused features on the PN filtering samples. During the evaluation, we used the filtered standalone and fused features-set to test the ensemble subspace discriminate classifier for ASV. We obtained an accuracy and precision of 0.96, recall of 0.96, an F1-score of 0.95, and an error rate of 0.04. In contrast with the comparative descriptors, we achieved error rates of 0.10, 0.10, 0.14, 0.08, 0.10, and 0.09 for TDoP, GTCC, MFCC, TDoP-GTCC, TDoP-MFCC, and MFCC-GTCC, respectively. PN filtering improved the accuracy of each descriptor on face masked signals but none of the existing features achieved better results than the proposed features. Although the difference in the error rate of the proposed features for audio samples (with and without mask) is high, the proposed PN filtering mitigates this deficiency using the TDoP features. Moreover, the proposed anomaly detection model achieved accuracy, precision, recall, and F1-scores of 0.99 for the classification of speech samples with and without face mask.

### 5.5. Performance Comparison Using Different Machine Learning Classifiers

The performance of the ASV systems relies on the front-end features, (i.e., cepstral/spectral, frequency, and phase-based features) as well as back-end classifiers used to determine the class of the speakers. In order to make a fair comparison, performance of the proposed system was evaluated against well-known Machine Learning (ML) classifiers. For this, we trained an ASV system with five ML classifiers including Decision Trees [41], K-Nearest Neighbor (KNN) [42], Naïve Bayes [43], Support Vector Machines (SVM) [44] and Linear Discriminant Analysis (LDA) [45]. All classifiers were tested on speech samples with and without face masks, and the results are listed in Table 4, Table 5, Table 6 and Table 7. For this experiment, we trained all classifiers using 80% speech samples recorded without face masks and tested against the remaining 20%. In addition, the ML classifiers were also tested against the speech samples with face masks.

In the first stage of this experiment, we measured the performance of our features on the decision trees classifier. In the decision trees experiment, we created decision trees with three different levels including fine, medium, and coarse distributions containing 100, 20, and 4 nodes, respectively. A Gini diversity index was used for the split criterion of the trees. The results are listed in Table 4, and show that the fine trees performed better than the medium and coarse trees. We achieved an error rate of 0.15, precision of 0.83, recall of 0.85, an F1-score of 0.84, and accuracy of 0.85. In contrast, we achieved an error rate of 0.43, and precision, recall, F1-score, and accuracy of 0.57 for the speech samples with face masks. These results reveal that the decision trees were unable to perform well for speaker verification due to the diversity in speech samples. Particularly, for the speech samples with face masks, the tree constructions (fine, medium, and coarse) produced a high error rate of 0.43, 0.52, and 0.81, respectively. Further, these results demonstrate that coarse trees using a smaller number of splits provided lower performance.

Next, we tested the effectiveness of the proposed features on an SVM classifier using the linear, quadratic, and cubic kernels. The results are reported in Table 5. For unmasked speech samples, We obtained the best results on the quadratic kernel, achieving an accuracy of 0.99. In contrast, for the masked speech samples, we achieved the best results (0.86 accuracy) on the linear kernel. The quadratic kernel performed well for face mask samples, however the performance dropped for masked speech samples. In contrast, the SVM with a linear kernel performed best on the face mask samples. Moreover, we used the level 1 box constraint and one vs. one multi-class method, and achieved the best results using these parametric settings. In the third stage, we evaluated our features on the KNN classifier. For this, we tested six distributions including fine, medium, coarse, cosine, cubic, and weighted KNN kernels. The results are presented in Table 6. We achieved a minimum error rate of 0.11 with the fine KNN kernel. The weighted distribution provided the second-best least error rate, 0.16, of all of the experiments. In contrast, coarse KNN produces the highest error rate of 0.77 and 0.79 for masked and unmasked speech samples. Finally, we tested the performance of our features on linear discriminant and Naïve Bayes classifiers. For Naïve Bayes, we examined the performance on the Gaussian and Kernel distributions. The results are reported in Table 7.

The Naïve Bayes distributions performed well for unmasked speech samples but performance declined drastically when the subjects wore different types of face masks. Particularly, we achieved an error rate of 0.12 and 0.10 with Gaussian and Kernel Bayes, respectively, on the unmasked samples. In contrast, 0.36 and 0.39 were achieved during the testing of masked speech samples. For linear discriminant, our features-set outperformed the state-of-the-art classifiers and provided the second-best results (after ensemble with subspace discriminant). Most significantly, the proposed system achieved error rates of 0.10 and 0.01 on the masked and unmasked samples. More precisely, a decrease of 0.09% in the precision, 0.10% in the recall, and 0.11% in the F1-score were observed in comparison with the unmasked testing. These scores in the statistical measurements prove the robustness of the proposed features with the linear discriminant classifier.

This experiment reveal that the linear discriminant classifier performs second-best, after the ensemble learner with subspace discriminant classifier. In addition, the results demonstrate that the proposed feature-set produced significant results within the linear nature of classifiers, i.e., Linear discrimination, ensemble learner with linear discriminant, and SVM with a linear distribution. In contrast, the classifiers with cubic and coarse distributions produce the highest error rates. It should be noted from the results that the performance of all ML classifiers showed a decline when tested with the masked speech samples. However, we overcome this limitation by applying the proposed anomaly detection and PN filtering solution in the ASV system.

### 5.6. Analysis of Multiple Face Masks on ASV Systems

The purpose of this experiment was to examine the effect of different face masks (surgical, cloth, and N95) on ASV systems. When confronted with several face masks, the results of five comparative ML-based and the proposed ensemble-based ASV classifiers reveal performance flaws in ASV systems. It is therefore important to keep track of how each face mask affects the ASV system. For this purpose, we conducted an experiment to examine how different face masks (surgical, cloth, and N95) influenced the ASV system. We tested the equal number of speech samples from each speaker and face mask. The results are mentioned in Table 8. These results demonstrate the degree of influence of various categories of face masks on ASV systems. The findings show that the ASV system severely misclassified the speech samples recorded using cloth masks. The proposed ASV system achieves the 0.93%, 0.94%, and 0.95% accuracies against cloth, N95, and surgical face masks, respectively. The results show that the ASV system severely misclassified the speech samples recorded using cloth face masks, with a 0.07 percent error rate. On the other hand, N95 was comparatively equal to the cloth face mask, and surgical face mask has the lowest impact with a lower error rate of 0.05%. Despite the fact that cloth masks have been found to be the most impacted face masks in the proposed ASV system, their influence is mostly dependent on fabric density.

### 5.7. Analysis of Different Distance and Face Masks on ASV systems

This experiment was performed to determine the impact of distance between the speaker and ASV system on verification. We analyzed the performance of the ASV system when the verification was performed from an approximate 45 cm and 90 cm distances, i.e., in a vehicular embedded microphone and subject. For this purpose, we tested the ASV system with speech samples of seven subjects recorded at distances of 45 cm and 90 cm between the subject and the microphone. In addition to the distance, we also tested the performance of the ASV system against speech samples recorded wearing different types of face masks (cloth, surgical, N95). We compared the performance of the ASV system with a close, 45 cm and 90 cm distant recording and the results are stated in Table 9. The results demonstrate that the performance of the ASV system is significantly degraded when the distance increases from close to approx 45 cm and 90 cm away. The error rate increased from 0.01 to 0.04 when we changed the distance up to 45 cm between the subject and the microphone. When the face masks speech samples were evaluated, the error rate of the ASV system increased from 0.08 to 0.12 and 0.14 against the 45 cm and 90 cm distances. From these results, it is notable that distance (with or without face masks) has a significant impact on ASV systems. Particularly, the performance of the ASV system dropped up to 7%, when the subjects used face masks from 90cm distance. However, PN filtering overcomes the performance deficiencies caused by distance. Our PN solution improved the error rate from 0.12 to 0.07 when testing face masked speech samples from 45cm and 0.15 to 0.06 against 90cm distance. In addition, the results stated in Table 9 demonstrate that the PN solution improves the ASV system performance up to 4% for unmasked users, and up to 8% against masked samples. For instance, accuracy of the system was increased from 0.92 to 0.96 in close-to-microphone testing and from 0.88 to 0.93 in 45cm and 0.85 to 0.94 in 90cm distance testing.

Hence, the results demonstrate the necessity of the PN solution in ASV systems. Further, it can be argued that under the existing COVID-19 circumstances, the performance of pre-trained ASV systems is negatively impacted when subject verification is performed from a specified distance (e.g., 45 cm and 90 cm in social distancing). However, the PN filtering overcomes the deficiencies of distance, as well as those caused by face masks, and can be used for the realignment of ASV systems without the need for retraining.

### 5.8. Analysis of Multiple Microphone and Face Masks on ASV Systems

This experiment was conducted to examine the impact of microphone type in automatic speaker verification. We analyzed the performance of ASV systems with different types of microphones (wired, wireless, cellphone). For this experiment, we tested the ASV system with speech samples of seven subjects, recorded with different microphones. The results of this experiment are reported in Table 10. It is worth discussing that the type of microphone performed an important role in automatic speaker verification and can adversely affect the ASV system, as revealed in this experiment. For instance, when we tested one-third of the subjects with different microphones than the ASV was trained, the error rate of the ASV system increased from 0.01 to 0.14. In addition to the changed microphones, when the subjects with different types of face masks were tested, the error rate increased up to 12%. However, with the PN solution, the error rate recovered to a considerable range of 0.13. More specifically, the accuracy increased from 0.80 to 0.87, precision and recall improved from 0.84 to 0.89, and the F1-score raised from 0.81 to 0.87. Consequently, this aspect of the analysis suggests that the performance of pre-trained ASV systems needs to be further analyzed, and the PN solution may be employed to improve the performance.

### 5.9. Analysis of Retraining of Asv Systems

According to the extensive experiments and the results reported in Table 4, Table 5, Table 6 and Table 7 the existing ASV systems failed to perform effectively when the speaker used multiple face masks, microphones, and authenticated from varied distances. The result, reported against five comparative ML classifiers, indicates a deficit in ASV system accuracy and reliability when confronted with multiple face masks. To observe the performance of the retrained ASV system, we carried out an experiment to see how well the ASV system performed after being retrained with the face masks speech sample. For this, we used speech samples recorded without face masks as well as three distinct face masks to train and test the ASV system. The results are reported in Table 11, and hybrid (with and without face masks) training and testing show that after retraining with face mask speech samples, the ASV systems’ accuracy improves significantly. The accuracy of the ASV system increased by 6%, from 0.92 to 0.98.

Although retraining the ASV system can be adopted to overcome face masks challenges, it is a time-consuming approach in terms of data collection and computational complexity. In addition, the massive amount of data (face mask speech samples from each speaker) required to retrain the ASV frameworks is, at best, problematic. Therefore, the finding demonstrates the necessity for the proposed cost-effective PN filtering method to eliminate the requirement for the retraining of the ASV systems. Moreover, the proposed solution also overcomes the need for additional data, along with its associated computations, and works sufficiently with multiple microphones and a wide range of distances.

We used Matlab 2020 for the implementation of the proposed and comparative methods. Furthermore, the proposed methods were implemented on an Intel(R) Core(TM) i5-5200U CPU @ 2.20 GHz system with 16 GB of memory.

## 6. Conclusions

According to the best of our knowledge, this research study is a first attempt to investigate the impact of the ensemble of type of face mask, microphone, and distance on ASV. The existing Gammatone and Mel cepstral coefficients are not sufficient to handle the added distortions when it comes to speaker verification. This paper presented a novel ASV framework to improve speaker verification capability without requiring users to give additional voice samples recorded with face masks. Experimentally, our proposed fusion of TDoP features, along with MFCC and GTCC coefficients, outperformed the standalone state-of-the-art descriptors. In addition, the proposed anomaly detection and PN solution optimally detects and recovers speech samples recorded with variations over face covering, microphone and distance. According to the findings of the experiments, the cloth mask has the most influence on the ASV system, the surgical mask has the least, and N95 has a moderate effect. Moreover, extensive experiments demonstrate a desperate need for the realignment of existing ASV systems. The improvement of accuracy from 0.92 to 0.96 with the PN solution signifies the effectiveness of the proposed framework for automatic speaker verification. In accordance with the methods proposed in this study, some computational intelligence algorithms, such as monarch butterfly optimization (MBO) [46], earthworm optimization algorithm (EWA) [47], colony predation algorithm (CPA) [48], and Harris hawks optimization (HHO) [49] may be used to enhance the efficiency of the algorithm.

In the future, we would like to extend the proposed framework for speech to text conversion and speech intelligibility. We also plan to evaluate the impact of face mask on ASV and voice spoofing countermeasures jointly [50], and on the single and multi-order voice replay spoofing countermeasure [51], and voice cloning detection [52]. 

## Figures and Tables

**Table 1:** Details of the Multi Speaker Face Mask (MSFM) dataset.

Audio	Mask Type	Devices	Subjects	Microphone	Distance	Samples
Without Mask	–	Single	20	Single	Close	200
Without Mask	–	Multiple	7	Multiple	Close	63
Without Mask	–	Single	7	Single	45 cm	63
Without Mask	–	Single	7	Single	90 cm	63
With Masks	N95, Cloth, Surgical	Single	20	Single	Close	240
With Masks	N95, Cloth, Surgical	Multiple	7	Multiple	Close	63
With Masks	N95, Cloth, Surgical	Multiple	7	Multiple	45 cm	63
With Masks	N95, Cloth, Surgical	Single	7	Single	90 cm	63

**Table 2:** Results of proposed method on different ensemble classifiers.

Classifier	Training	Testing	Err	Pr	Re	F1	Accuracy	Kappa	MCC	CSI
Bagged	0.91	Without Masks	0.10	0.90	0.89	0.89	0.90	0.70	0.90	0.94
Tree	–	With Masks	0.37	0.66	0.64	0.64	0.63	0.21	0.67	0.60
Subspace	0.70	Without Masks	0.70	0.30	0.31	0.31	0.30	0.84	0.23	0.23
KNN	–	With Masks	0.75	0.22	0.24	0.23	0.25	0.74	0.23	0.23
Subspace	0.99	Without Masks	0.01	0.99	0.99	0.99	0.99	0.98	0.99	0.98
Discriminant	–	With Masks	0.08	0.91	0.92	0.91	0.92	0.86	0.90	0.98
Rusboosted	0.73	Without Masks	0.27	0.73	0.73	0.72	0.73	0.73	0.71	0.70
Trees	–	With Masks	0.45	0.57	0.55	0.56	0.55	0.68	0.67	0.56
Boosted	0.95	Without Masks	0.01	0.97	0.98	0.99	0.95	0.68	0.99	0.99
Trees	–	With Masks	0.31	0.74	0.70	0.67	0.69	0.40	0.67	0.56

**Table 3:** Comparative analysis of the proposed and other spectral features with (a) TDoP (b) GTCC (c) MFCC (d) TDoP-GTCC (e) TDoP-MFCC (f) MFCC-GTCC (g) TDoP-MFCC-GTCC.

Features	Training	Testing	Err	Pr	Re	F1	Accuracy	Kappa	MCC	CSI
(a)	0.95	Without Masks	0.05	0.96	0.95	0.94	0.95	0.47	0.95	0.95
	–	With Masks	0.12	0.88	0.91	0.88	0.88	0.18	0.88	0.86
	–	Filtered Masks	0.10	0.89	0.90	0.89	0.90	0.20	0.89	0.87
(b)	0.94	Without Masks	0.10	0.90	0.91	0.89	0.90	0.32	0.89	0.88
	–	With Masks	0.15	0.86	0.85	0.85	0.85	0.03	0.85	0.78
	–	Filtered Masks	0.10	0.92	0.90	0.90	0.90	0.05	0.90	0.87
(c)	0.91	Without Masks	0.13	0.87	0.90	0.86	0.87	0.60	0.87	0.86
	–	With Masks	0.25	0.78	0.75	0.75	0.75	0.24	0.74	0.68
	–	Filtered Masks	0.14	0.86	0.86	0.86	0.86	0.30	0.85	0.78
(d)	0.97	Without Masks	0.03	0.98	0.97	0.97	0.97	0.73	0.97	0.97
	–	With Masks	0.12	0.89	0.88	0.88	0.88	0.07	0.89	0.82
	–	Filtered Masks	0.08	0.93	0.91	0.91	0.92	0.08	0.91	0.90
(e)	0.97	Without Masks	0.08	0.92	0.92	0.92	0.92	0.24	0.92	0.88
	–	With Masks	0.13	0.87	0.88	0.87	0.87	0.21	0.97	0.81
	–	Filtered Masks	0.10	0.91	0.89	0.89	0.90	0.08	0.89	0.86
(f)	0.96	Without Masks	0.05	0.96	0.95	0.95	0.95	0.12	0.94	0.95
	–	With Masks	0.12	0.88	0.88	0.88	0.88	0.10	0.88	0.82
	–	Filtered Masks	0.09	0.93	0.91	0.90	0.91	0.12	0.91	0.88
(g)	0.99	Without Masks	0.01	0.99	0.97	0.98	0.99	0.10	0.98	0.99
	–	With Masks	0.08	0.91	0.92	0.91	0.92	0.03	0.90	0.84
	–	Filtered Masks	0.04	0.96	0.96	0.90	0.96	0.47	0.94	0.92

**Table 4:** Verification results of decision trees.

Kernels	Training	Testing	Err	Pr	Re	F1	Accuracy	Kappa	MCC	CSI
Fine	0.79	Without Masks	0.15	0.83	0.85	0.84	0.85	0.24	0.84	0.84
	–	With Masks	0.43	0.57	0.57	0.57	0.57	0.73	0.62	0.52
Medium	0.73	Without Masks	0.28	0.73	0.72	0.72	0.72	0.01	0.73	0.72
	–	With Masks	0.52	0.49	0.47	0.48	0.48	0.79	0.50	0.48
Coarse	0.21	Without Masks	0.75	0.24	0.22	0.25	0.25	0.87	0.25	0.24
	–	With Masks	0.81	0.19	0.17	0.19	0.19	0.88	0.18	0.19

**Table 5:** Verification results of support vector machines.

Kernels	Training	Testing	Err	Pr	Re	F1	Accuracy	Kappa	MCC	CSI
Linear	0.97	Without Masks	0.02	0.97	0.98	0.97	0.98	0.73	0.97	0.97
	–	With Masks	0.14	0.85	0.86	0.85	0.86	0.34	0.85	0.78
Quadratic	0.97	Without Masks	0.01	0.96	0.98	0.97	0.99	0.10	0.98	0.98
	–	With Masks	0.16	0.84	0.84	0.84	0.84	0.34	0.85	0.78
Cubic	0.96	Without Masks	0.03	0.97	0.98	0.96	0.97	0.10	0.98	0.98
	–	With Masks	0.18	0.85	0.81	0.83	0.82	0.41	0.83	0.75

**Table 6:** Verification results of K-Nearest Neighbor (KNN).

Kernels	Training	Testing	Err	Pr	Re	F1	Accuracy	Kappa	MCC	CSI
Fine	0.96	Without Masks	0.01	0.97	0.98	0.99	0.99	0.99	0.40	0.99
	–	With Masks	0.11	0.86	0.88	0.89	0.89	0.12	0.88	0.83
Medium	0.86	Without Masks	0.10	0.94	0.90	0.90	0.90	0.45	0.90	0.95
	–	With Masks	0.19	0.80	0.82	0.80	0.81	0.60	0.74	0.70
Coarse	0.18	Without Masks	0.77	0.25	0.22	0.23	0.23	0.84	0.85	0.85
	–	With Masks	0.79	0.19	0.21	0.19	0.21	0.88	0.98	0.20
Cosine	0.86	Without Masks	0.10	0.92	0.90	0.90	0.90	0.36	0.90	0.93
	–	With Masks	0.24	0.75	0.77	0.75	0.76	0.66	0.70	0.64
Cubic	0.87	Without Masks	0.10	0.92	0.90	0.89	0.90	0.36	0.90	0.92
	–	With Masks	0.21	0.81	0.78	0.77	0.79	0.62	0.73	0.67
Weighted	0.96	Without Masks	0.01	0.97	0.98	0.99	0.99	0.40	0.99	0.99
	–	With Masks	0.16	0.85	0.84	0.83	0.84	0.41	0.83	0.76

**Table 7:** Performance analysis on the naïve bayes and linear discriminant.

Kernels	Training	Testing	Err	Pr	Re	F1	Accuracy	Kappa	MCC	CSI
Gaussian	0.95	Without Masks	0.12	0.87	0.89	0.87	0.88	0.05	0.89	0.87
Naïve	–	With Masks	0.36	0.65	0.64	0.65	0.64	0.74	0.69	0.67
Kernel	0.93	Without Masks	0.10	0.90	0.91	0.90	0.90	0.24	0.98	0.90
Naïve	–	With Masks	0.39	0.61	0.63	0.62	0.61	0.74	0.62	0.65
Linear	0.99	Without Masks	0.01	0.99	0.99	0.99	0.99	0.73	0.97	0.98
Discriminant	–	With Masks	0.10	0.90	0.89	0.88	0.90	0.16	0.91	0.86

**Table 8:** Comparative analysis of multiple face masks.

Face Mask	Err	Pr	Re	F1	Accuracy	Kappa	MCC	CSI
Surgical	0.05	0.96	0.95	0.94	0.95	0.34	0.93	0.95
Cloth	0.07	0.93	0.92	0.93	0.93	0.05	0.89	0.92
N95	0.06	0.94	0.93	0.94	0.94	0.34	0.93	0.91

**Table 9:** Comparative analysis of close and distance samples.

Distance	Testing	Err	Pr	Re	F1	Accuracy	Kappa	MCC	CSI
Close	Without Masks	0.01	0.99	0.99	0.99	0.99	0.99	0.99	0.98
	With Masks	0.08	0.91	0.92	0.91	0.92	0.86	0.90	0.90
	Filtered Masks	0.04	0.95	0.96	0.95	0.96	0.88	0.95	0.94
45 cm away	Without Masks	0.04	0.98	0.98	0.98	0.96	0.35	0.96	0.98
	With Masks	0.12	0.88	0.89	0.87	0.88	0.30	0.87	0.85
	Filtered Masks	0.07	0.93	0.93	0.93	0.93	0.33	0.93	0.90
90 cm away	Without Masks	0.04	0.98	0.98	0.98	0.96	0.84	0.85	0.93
	With Masks	0.15	0.86	0.85	0.86	0.85	0.38	0.85	0.82
	Filtered Masks	0.06	0.94	0.94	0.93	0.94	0.38	0.93	0.90

**Table 10:** Comparative analysis of trained and changed microphones.

Microphone	Testing	Err	Pr	Re	F1	Accuracy	Kappa	MCC	CSI
Trained	Without Masks	0.01	0.99	0.97	0.98	0.99	0.10	0.95	0.99
	With Masks	0.10	0.91	0.92	0.91	0.92	0.08	0.90	0.84
	Filtered Masked	0.04	0.95	0.96	0.95	0.96	0.47	0.94	0.92
Changed	Without Masks	0.14	0.90	0.89	0.85	0.86	0.10	0.87	0.88
	With Masks	0.20	0.84	0.84	0.81	0.80	0.12	0.80	0.81
	Filtered Masked	0.13	0.89	0.89	0.87	0.87	0.34	0.86	0.90

**Table 11:** Comparative analysis of trained and retrained ASV system.

ASV	Training	Testing	Err	Pr	Re	F1	Accuracy	Kappa	MCC	CSI
Trained	Without Mask	Without Masks	0.01	0.99	0.99	0.99	0.99	0.98	0.99	0.97
	Without Mask	With Masks	0.08	0.91	0.92	0.91	0.92	0.03	0.90	0.84
Retrained	Hybrid	Hybrid	0.02	0.98	0.98	0.98	0.98	0.99	0.99	0.98

## Data Availability

The datasets, libraries, and any supporting tools used or analyzed during the current work are accessible upon reasonable request from the corresponding author.
